# Effect of distillery yeast sludge on growth performance, nutrient digestibility and slaughter parameters in Japanese quails

**DOI:** 10.1038/s41598-018-26741-6

**Published:** 2018-05-30

**Authors:** M. Sharif, M. Shoaib, M. Aziz Ur Rahman, F. Ahmad, Shahid Ur Rehman

**Affiliations:** 1 Institute of Animal and Dairy Sciences, University of Agriculture, Faisalabad, Pakistan; 2 Sub-campus, Toba Tek Singh, University of Agriculture, Faisalabad, Pakistan

## Abstract

This study was planned to evaluate the effect of distillery yeast sludge (DYS) as a protein source on growth performance, nutrient digestibility and slaughtering parameters in Japanese quail birds. For this purpose, six hundred day-old quails were randomly distributed into six dietary treatments. These dietary treatments were; G_1_ (Control Group) fed a basal diet (CP 24%, ME 2900 kcal/kg) without DYS, while other dietary treatments were G_2_, G_3_, G_4_, G_5_ and G_6_ fed basal diet containing DYS @ 1.5, 3.0, 4.5, 6.0 and 7.5%, respectively. It was observed that feed intake and weight gain tended to increase (P < 0.05) up to 3% DYS. Better (P < 0.05) feed conversion ratio (FCR) was observed in birds fed diet containing DYS at the level of 1.5 and 3%. Dry matter and crude protein digestibility decreased with increasing levels of DYS (P < 0.05). Cost of production per 100 g of weight gain was also less in birds fed diet containing 3% DYS. Study revealed that inclusion of distillery yeast sludge in the diet of Japanese quails up to 3% improves the growth performance and economics efficacy.

## Introduction

Chicken is one of the major source of protein for human but efforts are being made for exploiting other suitable economical sources. Quail meat is another good source of animal protein. Japanese quail (*Coturnix coturnix japonica*) has high resistance to diseases and can be used for meat production due to short market age^1^.

Nutrition is backbone of poultry farming because it contributes 65 to 70% of the total production cost^2^. Natural sources like soybean meal and canola meal are being used to meet the bird’s protein requirements. However, their lesser availability and higher prices necessitate for the use of other alternative cheaper non-conventional protein sources. Several microbes like algae, bacteria, yeast and fungus act as protein producers^3^ and could be used in the diet of poultry. Of all the microbes, yeast is mainly used in the production of single cell protein. This is because of its rapid growth rate and high efficiency to convert carbon sources into protein^4^. Yeast and yeast products produced from agro-industrial by products are rich in protein contents^5^. Molasses fermented by *Saccharomyces*^6^ and its waste products can be used in the diet of poultry. Yeast sludge (product of distillery industry) contains 27–29% crude protein^7^ and can be used in poultry diet^8^. It also contains some essential amino acids which are necessary for proper growth and health of birds^9^.

Distillery yeast sludge (DYS) refers to surplus yeast at the bottom of fermentation tank in the form of sludge during the fermentation process of sugar and distillery industries. It is also named as spent yeast, yeast slurry and trub. It is considered as a waste product and it is very difficult to manage and dispose it^10^. In Pakistan, about 1,300 tons DYS is produced and wasted annually^11^. However, it can be used in the diet of poultry. There is not enough evidence about its use in Japanese quails, therefore, this research was planned to study the effect of distillery yeast sludge as a protein source on growth performance, nutrient digestibility, economics and slaughter parameters in Japanese quails.

## Results

Results were compared on diets containing DYS at the level of 0, 1.5, 3, 4.5, 6 and 7.5% (Table 1).

**Table 1:** Ingredients and nutrients composition of experimental diets.

Ingredients	Experimental diets					
	G1	G2	G3	G4	G5	G6
Corn	41.44	38.61	37.60	36.44	35.27	34.10
Rice tips	10.07	12.00	12.00	12.00	12.00	12.00
Rice polish	2.00	2.00	2.00	2.27	2.55	2.82
Corn gluten 60%	3.00	3.00	3.00	3.00	3.00	3.00
Soyabean meal	23.54	22.96	22.08	21.52	20.97	20.44
Canola meal	5.00	5.00	5.00	5.00	5.00	5.00
Sunflower meal	3.00	3.00	3.00	3.00	3.00	3.00
Fishmeal	3.00	3.00	3.00	3.00	3.00	3.00
Guar meal	1.50	1.54	1.99	2.00	2.00	2.00
Poultry by product meal	2.00	2.00	2.00	2.00	2.00	2.00
Vegetable oil	1.50	1.50	1.50	1.50	1.50	1.50
Molasses	1.00	1.00	1.00	1.00	1.00	1.00
Limestone	0.46	0.43	0.41	0.39	0.37	0.35
DCP	1.45	1.42	1.37	1.33	1.29	1.24
Vit. min premix	0.50	0.50	0.50	0.50	0.50	0.50
L-Lysine	0.30	0.31	0.32	0.33	0.34	0.35
DL-Methionine	0.17	0.16	0.16	0.15	0.15	0.14
L-Threonine	0.07	0.07	0.07	0.07	0.06	0.06
Yeast sludge	0.00	1.50	3.00	4.50	6.00	7.50
**Total**	**100**	**100**	**100**	**100**	**100**	**100**
**Nutrients**	**G1**	**G2**	**G3**	**G4**	**G5**	**G6**
Crude protein (%)	24.00	24.00	24.00	24.00	24.00	24.00
Metabolizable Energy (Kcal/Kg)	2900.01	2900.13	2900.01	2899.95	2899.83	2899.91
Crude fiber (%)	3.55	3.58	3.66	3.72	3.78	3.85
Lysine (%)	1.30	1.30	1.30	1.30	1.30	1.30
Methionine (%)	0.55	0.55	0.55	0.55	0.55	0.55
Threonine (%)	0.85	0.85	0.85	0.85	0.85	0.85
Ca (%)	0.90	0.90	0.90	0.90	0.90	0.90
Av. P (%)	0.48	0.48	0.48	0.48	0.48	0.48

### Body weight gain

Weight gain was 13.61% higher (P < 0.05) in birds fed diet containing 3% DYS compared to birds fed diet containing 7.5% DYS (Table 2). It was 4.59% more in birds fed 3% DYS as compare to control.

**Table 2:** Values of feed intake, body weight gain and feed conversion ratio per quail chick fed diets containing DYS.

Parameters	Dietary Treatments							
	Control	1.5% DYS	3.0% DYS	4.5% DYS	6.0% DYS	7.5% DYS	SEM	P value
**Starter phase (day 1–21)**								
Feed intake (g)	207.11^a^	209.05^a^	210.28^a^	203.15^a^	199.58^b^	194.21^c^	2.65	*
Weight gain (g)	78.67^abc^	81.84^ab^	82.88^a^	76.81^bc^	74.33^c^	68.77^d^	1.69	***
Feed conversion ratio	2.63	2.56	2.54	2.65	2.69	2.83	0.07	NS
**Finisher phase (day 22–35)**								
Feed intake (g)	280.74^ab^	283.43^a^	285.24^a^	277.29^bc^	273.71^c^	268.84^d^	1.59	***
Weight gain (g)	63.74^b^	66.27^a^	66.37^a^	62.87^b^	62.76^b^	60.17^c^	0.77	***
Feed conversion ratio	4.40	4.27	4.29	4.41	4.36	4.47	0.06	NS
**Overall period (day 1–35)**								
Feed intake (g)	487.85^ab^	492.49^ab^	495.53^a^	480.44^ab^	473.29^bc^	463.95^c^	3.45	***
Final body weight (g)	149.75^bc^	155.40^ab^	156.48^a^	146.97^c^	144.30^c^	136.15^d^	1.91	***
Weight gain (g)	142.41^b^	148.11^a^	149.26^a^	139.69^b^	137.11^b^	128.94^c^	1.87	***
Feed conversion ratio	3.42^ab^	3.33^b^	3.32^b^	3.44^ab^	3.45^ab^	3.59^a^	0.06	*
Mortality Rate (No.)	1	1	1	2	1	3		

Values with different super-scripts (a, b, c and d) are significantly (P < 0.05).

NS: non-significant (P > 0.05); *P < 0.05; **P < 0.01; ***P < 0.001.

### Feed intake

Feed intake was highest (P < 0.05) in birds fed diet containing 1.5 and 3.0% DYS and lowest in birds fed diet containing 7.5% DYS (Table 2). Birds fed diet containing 3% DYS consumed 6.37% more feed compare to birds fed 7.5% DYS.

### Feed conversation ratio

In starter and finisher phase, feed conversion ratio (FCR) was non-significantly different. Overall, better (p < 0.05) FCR was observed in birds fed diet containing DYS at the level 3.0%, whereas, poor FCR was observed in birds fed diet containing DYS at the level of 7.5% (Table 2).

### Digestibility

**Figure 1:**
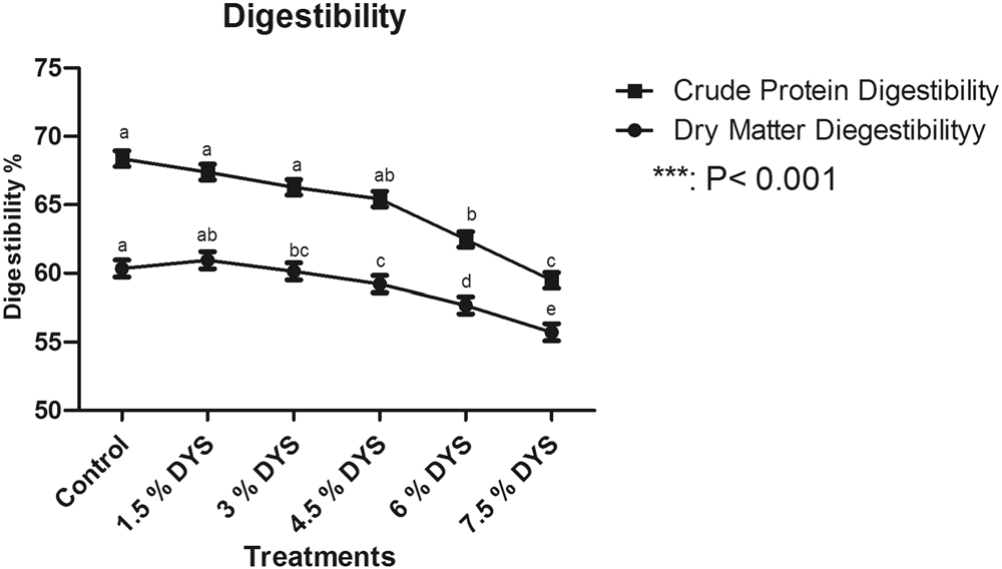
Nutrients digestibility of quail birds fed diets containing different levels of DYS ***P < 0.001.

Dry matter digestibility decreased with increasing the level of yeast sludge above 3%. It was higher in birds fed control, 1.5 and 3.0% and lower in birds fed diet containing 7.5% DYS. Crude protein digestibility was also decreased with increasing levels of DYS (p < 0.05) (Fig. 1).

### Carcass characteristics

Carcass characteristics (dressing percentage, breast meat yield and thigh meat yield) and relative organs weight (liver, heart and gizzard) were not significantly different (P > 0.05) in birds fed diets containing various levels of DYS (Table 3).

**Table 3:** Average values of dressing percentage and breast meat, thigh meat and giblet organs.

Slaughter parameters (%)	Dietary treatments							
	Control	1.5% DYS	3.0% DYS	4.5% DYS	6.0% DYS	7.5% DYS	SEM	P value
Dressing percentage	57.46	56.65	57.71	55.34	55.34	56.22	0.78	NS
Breast meat weight	37.52	36.67	37.25	37.49	36.98	36.30	0.57	NS
Thigh meat weight	19.94	19.99	20.46	19.70	18.35	19.93	0.78	NS
Heart weight	0.90	0.89	0.91	0.88	0.92	0.89	0.02	NS
Gizzard weight	2.48	2.51	2.63	2.54	2.65	2.93	0.06	NS
Liver weight	2.79	2.76	2.84	2.82	2.98	2.94	0.08	NS

NS: non-significant (P > 0.05).

### Economics

**Figure 2:**
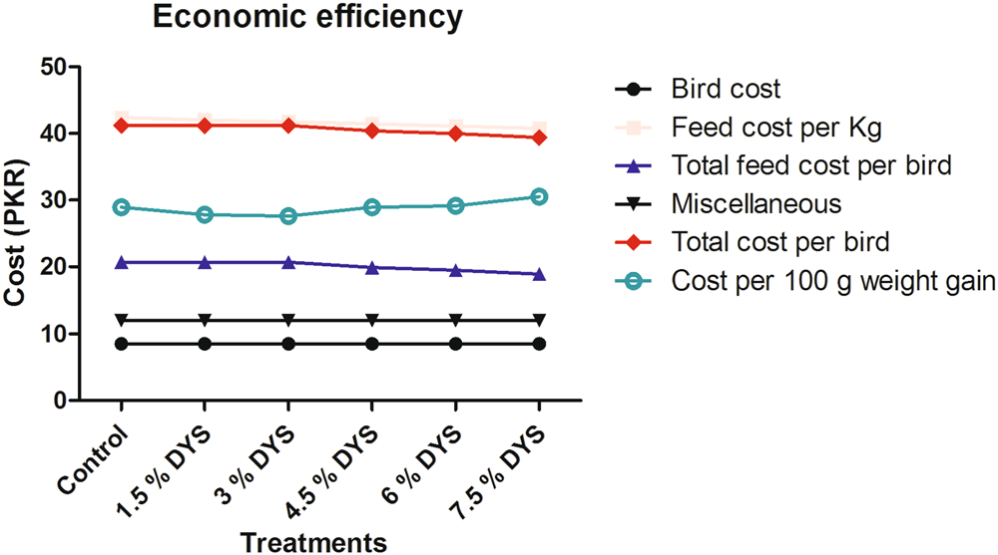
Economic efficiency feeding diets containing different levels of DYS in quail birds.

The economics of feeding diets containing different levels of DYS indicated that cost of production per 100 g of weight gain was reduced by increasing the inclusion of DYS up to 3% (Fig. 2).

## Discussion

Significant improvement in weight gain was observed in birds fed diet having 3%DYS. The possible reason of improved weight gain was due to higher feed intake and biological value of protein present in DYS. Machalek, *et al*.^12^ reported that replacing soybean meal with equal amount of brewer’s yeast having 48% CP resulted in improved weight gain. Other researchers^13–16^ found that inclusion of yeast/ distillery sludge yeast in poultry diets improved weight gain. Reduced weight gain at higher levels (6 and 7.5%) of DYS was due to lower feed intake which was associated with higher level of nucleic acids^16,17^ and poor digestibility of DYS^16^. Sharif, *et al*.^16^ reported significant improvement in broiler live weight gain as the level of DYS was increased up to 8% while it decreased at higher level of DYS i.e. 12%. Similar findings were observed by Khan^11^ who reported that increasing the level of DYS up to 6% improved weight gain in birds while 9% DYS had adverse effect on live weight gain. In another study, it was found that inclusion of distillery sludge up to 5% in bird’s diet has no detrimental effects while increasing the level up to 10% retarded growth^18^.

Feed intake was higher (P < 0.05) in birds fed diet containing 1.5 and 3.0% DYS and lower in birds fed diet containing 7.5% DYS (Table 2). These results are in line with the findings of Sharif, *et al*.^16^ who reported that feed intake was increased by the addition of DYS in broiler diet. The higher feed intake in birds fed diet containing 1.5 and 3.0% DYS was possibly due to the fact that distillery sludge is a rich source of vitamins especially B-complex, oligomonosaccharides (natural aflatoxin binder) and unidentified nutrients, all of which might have contributed to increase the palatability of diet^16,19^. If distillery sludge is a rich source of vitamins especially B-complex, oligomonosaccharides (natural aflatoxin binder) and unidentified nutrients, birds fed diet containing 7.5% DYS should have higher feed intake. However, in the present study, the opposite results were observed. Oliva-Teles, *et al*.^17^ also reported decreased feed intake in birds fed diet containing 12% distillery sludge. Our findings are in consistent with the findings of previous studies who reported decline in feed intake with increasing levels of brewer’s dried yeast^9,20^. Despite of rich source of vitamins, oligomonosaccharides and unidentified nutrients distillery sludge contains nucleic acids which are the major reason of reduced feed intake.

Non-significant difference of FCR in starter and finisher phase, and overall better FCR in diet containing DYS at the level of 3.0% were in consistent with our previous findings^16^. Sharif, *et al*.^16^ reported non-significant differences in FCR among diets containing different levels of washed distillery yeast. Kahraman, *et al*.^21^ also reported non-significant difference among treatment groups supplemented with organic acid (acid dry 3 g/kg), yeast culture (yea sacc1026 1 g/kg) and organic acid and yeast culture (acid lac dry 3 g/kg and yea sacc 1026 1 g/kg), respectively. Contrary to these, Nilson, *et al*.^14^ found better FCR in birds fed yeast containing diets. Khan^11^ also reported that FCR was significantly improved in diets containing 6% RDS. Similar findings were also reported by other researchers^14,22^. Improved FCR is due to the reason that distillery sludge is a rich source of vitamins, oligomonosaccharides, and some unidentified nutrients, which contributed better performance of the birds.

Dry matter digestibility decreased with increasing the level of yeast sludge above 3%. Crude protein digestibility was also decreased with increasing levels of DYS. It was higher (p < 0.05) in birds fed control diet and lower in birds fed diet containing 7.5% DYS. The decrease in dry matter and protein digestibility with increasing the level of the DYS in the diet showed the superiority of the control diet over other treatment diets. This therefore, implies that the birds on control diet had better crude protein and dry matter digestibility. Similar findings were reported by^23,24^. However, the similarity between the birds on the control diet, and those on 3% DYS in the proportion of dry matter digestibility suggested that DYS may be incorporated up to 3% in quails diets without deleterious effects on dry matter digestibility. However the decrease of crude protein digestibility at 3% DYS inclusion may be due to better amino acid profile of soybean meal in the control diet.

Carcass characteristics (dressing percentage, breast meat yield and thigh meat yield) and relative organs weight (liver, heart and gizzard) were not significantly different (P > 0.05) in birds fed diets containing various levels of DYS. Although there were some apparent differences in dressing percentage and other parameters, however, statistically these differences were non-significant. Findings of Sharif *et al*. (2012) are also in alignment with present study in which they observed non-significant difference on carcass characteristics in birds fed 8, 12 and 16% DYS in broiler diets. However^25^, reported that yeast has significant effect on dressing percentage and breast meat in broiler chicks.

The economics of feeding diets containing different levels of DYS indicated that cost of production per 100 g of weight gain was reduced by increasing the inclusion of DYS up to 3%. These results are in line with Chirwa and Lebitso^26^ who revealed that growing chicken on preprocessed waste activated sludge saved the cost due to the fast growth rate. Results are also in line with the findings of Rameshwari and Karthikeyan^9^ and Sharif *et al*. (2016) who concluded that feed cost was reduced due to addition of DYS in the diet. This might be attributed to cheaper availability of DYS that resulted in cost effective ration formulation.

## Conclusion

Feeding increasing levels of distillery yeast sludge in the quail’s diet resulted in improved weight gain, feed intake and feed conversion ratio. However, inclusion of distillery yeast sludge up to 3% showed better results in terms of weight gain and economics. Dressing percentage remained unaffected by feeding sludge in the diet. This indicates that distillery yeast sludge can be included in the quail’s diet up to 3% without any deleterious effect on the performance of the quail birds.

## Materials and Methods

Six iso-caloric (ME 2900 kcal/kg) and iso-nitrogenous (CP 24%) diets were formulated containing DYS at the level of 0, 1.5, 3, 4.5, 6 and 7.5% (Table 1). Diets were formulated according to the nutrient requirement of quail (NRC, 1994). The DYS procured from Shakhargung Sugar Mills Ltd., Jhang. Before inclusion in the diet DYS was washed at the rate of 6:1 (water:sludge) as described by Sharif *et al*., 16. For this, 150 kg DYS was washed by adding 900 kg water. The material was placed unmoved for 8 h, which allowed the biomass to settle at the base. After this, water collected above the biomass was removed simply by tilting the container. The biomass remained after washing and removal of water was sun dried. One hundred and fifty square feet floor space was washed to remove dust and the biomass was poured over the area for drying. The flakes of dried DYS were removed by using scraper and ground in the hammer mill to a mash size of 2 mm. That grounded DYS was included in the diet. The acid insoluble ash (Celite®) was used as digestibility marker and was added at 1% of the feed. All feed ingredients used to formulate diets were purchased from local market and feed was formulated at University of Agriculture, Faisalabad feed mill. Experimental diets were offered ad libitum in mash form.

The quails were managed according to the principles and specific guidelines of the Institute of Animal and Dairy Sciences, University of Agriculture Faisalabad. All of the procedures were approved by the scrutiny committee of Institute of Animal and Dairy sciences, University of Agriculture Faisalabad. The experiment was approved by the Head of Institute of Animal and Dairy sciences, University of Agriculture Faisalabad. Six hundred day-old Japanese quail chicks (*Coturnix coturnix japonica*) were purchased from local hatchery. Chicks were randomly distributed to six dietary treatments, 100 in each group. Dietary treatments included G_1_, G_2_, G_3_, G_4_, G_5_ and G_6_. Each treatment was further subdivided into four subgroups in order to make replicates, each containing 25 quails. Each treatment group was placed in separate pen (4ʹ × 2.5ʹ × 2.5ʹ). Experimental birds were reared on floor litter system; a layer of 2 inch of saw dust was used as bedding material which was evenly spread throughout the pens. Bedding material was covered with corrugated paper to avoid the injury to the feet of quail birds. Temperature was maintained at 95 F̊ during the first week of brooding period and was gradually decreased by 5 F̊ every day until its reached a temperature of 75 F̊ during the 5^th^ week of age. Data on feed intake, body weight gain and mortality were recorded and feed conversion ratio (FCR) was calculated.

On day 32 of the trail, digestibility was carried out. Acid insoluble ash mixed diet were fed to the quails from day 32 to 34. Polytene sheets were placed under each pen. Acid insoluble of feed was also measured. These excreta samples within the pen were pooled, weighed, homogenized, and oven dried in a hot air oven at 65 °C and ground to pass through 0.5 mm sieve. These were then kept at −10 °C until further analyses. Diets and excreta samples were subjected to chemical analysis (AOAC, 2000).

Economics of production was also calculated. At the end of study, two birds from each replicate were selected randomly and slaughtered to obtain data on carcass characteristics (dressing percentage, breast meat yield and thigh meat yield) and relative organs weight (liver, heart, gizzard).

Data were analyzed using analysis of variance technique by completely randomized design (CRD) using SAS and Duncan’s multiple range test (DMR) was used to compare the means.
